# Physiological Comparisons of Elite Male Visma Ski Classics and National Level Cross-Country Skiers During Uphill Treadmill Roller Skiing

**DOI:** 10.3389/fphys.2018.01523

**Published:** 2018-11-16

**Authors:** Edvard H. Sagelv, Tina P. Engseth, Sigurd Pedersen, Svein A. Pettersen, Gunnar Mathisen, Kim A. Heitmann, Boye Welde, Tor O. Thomassen, Thomas L. Stöggl

**Affiliations:** ^1^School of Sport Sciences, Faculty of Health Sciences, UiT The Arctic University of Norway, Tromsø, Norway; ^2^Department of Teaching and Pedagogy, Faculty of Humanities, Social Sciences and Teaching, UiT The Arctic University of Norway, Tromsø, Norway; ^3^Department of Sport and Exercise Science, University of Salzburg, Salzburg, Austria

**Keywords:** diagonal stride, double poling, long-distance, gross efficiency, maximal oxygen uptake, submaximal oxygen uptake

## Abstract

Longer distance cross-country ski (14–220 km) races such as the Visma Ski Classics (VSC) has recently gained attention in addition to the traditional Olympic distances (5–50 km) associated with cross-country (XC) skiing. These long-distance races are characterized by extensive use of the upper body while double poling (DP). While there is a substantial amount of research on Olympic distance XC skiing, the physiological capacities of VSC skiers has not yet been explored. We recruited seven elite male VSC skiers and seven well-trained national level male XC skiers to undergo three tests in the laboratory: (1) a one repetition maximum (1RM) strength test in a cable pulldown; (2) roller skiing tests on a treadmill (10.5% inclination) for determination of gross efficiency (GE) at submaximal speeds (8 and 10 km·h^−1^) in DP and diagonal stride (DS); (3) two ramp protocols to exhaustion (15% inclination, starting speed 7 km·h^−1^) in DP and DS for the assessment of peak and maximal oxygen uptake ($\dot{\text{V}}$O_2peak_ and $\dot{\text{V}}$O_2max_), respectively. Compared with the national level XC skiers, the VSC skiers performed similar in the 1RM cable pulldown, displayed 12.2% higher GE in DP at 8 km·h^−1^ but did not display any difference at 10 km·h^−1^, and had lower blood lactate concentration and heart rate at both submaximal speeds. The VSC skiers had longer time to exhaustion compared with the national level XC skiers during the two ramp protocols in DS (18%) and in DP (29%). The $\dot{\text{V}}$O_2max_ was 10% higher in DS compared with DP, with no differences between the groups. The $\dot{\text{V}}$O_2peak_/$\dot{\text{V}}$O_2max_-ratio of 90% did not differ between the two groups. In conclusion, the main differences were lower cardiorespiratory and metabolic responses at submaximal speeds as well as longer time to exhaustion in VSC skiers compared with national level XC skiers. This suggest efficiency to be the main difference between VSC and national level XC skiers.

## Introduction

Cross-country skiing (XCS) is unique due to its high demands for endurance and complex regulation and fluctuation of oxygen (O_2_) delivery for both the upper and lower body (Holmberg et al., 2007). Cross-country (XC) skiers compete in various forms and distances with the International Ski Federation (FIS) hosting the Olympic distances in their World Cup series. This series includes repetitive sprint competitions (1–3 km) and races over longer distances (5–50 km) in mass- and individual starts (Sandbakk and Holmberg, 2017). Lately, longer distance races (14–220 km, all mass start), such as the Wordloppet, Euroloppet, and the Visma Ski Classics (VSC) series, seem to gain more attention from both the skiers, public and media (Sandbakk and Holmberg, 2017). The VSC merges the most traditional long distance XCS events in Europe and worldwide, and the skiers only compete in the classic technique, compared with the FIS world cup series where also the skating technique is employed.

While there is a substantial amount of research upon XC skiers competing in Olympic distances, to the best of our knowledge, no study has yet focused on the physiological capacities of skiers performing in the popular long-distance races. The existing literature on long-distance skiing had a pure focus on the aspects of sex, age and performance level on pacing and performance (based on race times) during selected long-distance races (Nikolaidis and Knechtle, 2017, 2018a,b; Knechtle and Nikolaidis, 2018; Nikolaidis et al., 2018). Some physiological variables important to Olympic distance XCS performance can also be assumed to be important for long-distance XCS performance. It has been demonstrated that XC skiers display outstandingly high maximal oxygen uptake ($\dot{\text{V}}$O_2max_) (Åstrand, 1955; Saltin and Åstrand, 1967; Ekblom and Hermansen, 1968; Strømme et al., 1977; Bergh et al., 1978; Ingjer, 1991; Rusko, 1992; Holmberg et al., 2007; Tønnessen et al., 2015), and world-class skiers usually show higher $\dot{\text{V}}$O_2max_ compared with lower level skiers (Sandbakk et al., 2010). Further, as one would expect $\dot{\text{V}}$O_2max_ to be important in races over longer distances (>10 km) (Sandbakk and Holmberg, 2017), it appears that strength and peak speed is decisive for in sprint XCS performance (Stöggl et al., 2007, 2011; Sandbakk et al., 2011a; Østerås et al., 2016). As XCS techniques involve the upper body to various extents, in addition to a high aerobic power, the ability to perform on high rates close to their maximal aerobic power when mainly employing the upper body is considered decisive for XCS performance (Sandbakk and Holmberg, 2017). Finally, other physiological characteristics, such as work economy and/or efficiency has been suggested to be important for XCS performance (Stöggl et al., 2007; Sandbakk et al., 2010, 2011b; Østerås et al., 2016).

The diagonal stride (DS) has been the preferred sub-technique of choice in main parts of a classic XCS track (e.g., moderate to steep uphills). However, the use of the double poling (DP) sub-technique has evolved dramatically in recent years (Holmberg et al., 2006; Stöggl and Holmberg, 2011, 2016a; Holmberg, 2015). Until the recent introduction of “no DP” zones by FIS, several elite skiers exclusively used the DP sub-technique successfully throughout an entire race instead of using grip-wax (Holmberg et al., 2005; Stöggl and Holmberg, 2011, 2016a; Sandbakk and Holmberg, 2014; Welde et al., 2017; Stöggl et al., 2018). This development started in popular long-distance races consisting primarily of flat sections with slight climbs, and coincided with the introduction of the XCS sprint races. Alongside increased DP use, the maximal aerobic power of upper body musculature has improved dramatically, from 70% utilization of their whole body $\dot{\text{V}}$O_2max_ in the 1960s to 90% in the 1990s (Saltin, 1997). In the VSC series, currently all races (for both males and females), except the very demanding “Reistadløpet,” are won by using the DP sub-technique exclusively. Due to the marked development and utilization of the DP sub-technique, further investigations about the capacities of the present world-class VSC skiers is applicable.

Thus, the aim of this study was to examine the gross efficiency (GE), $\dot{\text{V}}$O_2max_ (in DS) and peak oxygen uptake ($\dot{\text{V}}$O_2peak_) (in DP) in male elite VSC skiers, as well as their one repetition maximum (1RM) in an upper body cable pulldown. A second aim was to compare these capacities to a group of well-trained national level male XC skiers.

## Methods

### Participants

Following the “Reistadløpet” of the VSC in April 2017, seven elite VSC male skiers, including the winner of the race, and seven national level XC skiers volunteered to participate in this study. The skiers' anthropometric characteristics, as well as performance level and training data, are shown in Table 1. All skiers were informed about the purpose of the study (verbally and in written text) before giving their written informed consent to participate. The study was pre-approved by the Norwegian Social Science Data Services and carried out in accordance with current ethical standards for sports and exercise research in accordance with the Declaration of Helsinki.

**Table 1:** Anthropometrics, performance level and training data of the elite Visma Ski Classics skiers (*n* = 7) and national level cross-country skiers (*n* = 7).

**Parameter**	**VSC**	**XC**
Age (yr)	29 (5)^*^	22 (2)
Height (cm)	180 (8)	178 (11)
Body mass (kg)	74.5 (10.6)	78.4 (18.4)
BMI (kg·m^−2^)	24.1 (0.8)	23.2 (3.9)
VSC-points	491 (1029)	N.A.
FIS distance-points	50 (104)	121 (68)
Total amount of training (h)	765 (253)^*^	660 (200)
DP-training (% of total)	53 (36)^***^	15 (11)

Data are shown as median (inter-quartile range). VSC, Visma Ski Classics skiers; XC, national level cross-country skiers; VSC-points, points obtained in the Visma Ski Classic tournament; DP-training, percentage double-poling of total amount of training.

* Significant difference between groups,^*^p < 0.05;

*** *p < 0.001*.

### Overall design of the study

On one testing day in the laboratory, the participants performed a 1 repetition maximum (1RM) test in a pulldown exercise, and roller skiing tests on a treadmill for determination of gross efficiency (GE) at submaximal speeds in DP and DS, in randomized order, followed by two ramp protocols to exhaustion with DP and DS for the assessment of $\dot{\text{V}}$O_2peak_ and $\dot{\text{V}}$O_2max_, respectively. For each skier, it took 2 h to complete all testing. The skiers consumed 300 ml of water following each separate test.

### Test concepts and instruments

All participants arrived at the laboratory in a rested and well-hydrated state, at least 2 h postprandial with no alcohol, caffeine and strenuous exercise 24 h prior to the tests, and with instructions to prepare as they would do for competition. Prior to testing, body mass and height were measured with a portable scale (Seca 876, Seca GmbH & Co. KG, Germany) and a stable stadiometer (Seca 217, Seca GmbH & Co. KG, Germany). Body mass index (BMI) was calculated. Following the body mass and height measurements, the skiers warmed up by running for 15 min on a self-selected speed (8–12 km·h^−1^, 0% and 5.3% inclination for 7.5 min each) on a motorized treadmill (ELG Woodway GmbH, weil an Rhein, Germany). The skiers reported their overall training and volume of DP-training based on their own training diary.

#### One repetition maximum pulldown test

Following the warm-up, the skiers performed a maximal strength test in a cable pulldown (Gymso, XMark Fitness, LA, USA), as previously described by Hegge et al. (2015). In order to isolate the upper body and exclude any possibility of employment of lower body musculature, the skiers were strapped around the hips while sitting on an adjustable bench in front of the cable pulldown with an approximately 90° angle at the knees, and with a stable back at approximately 120° angle to the seat. Prior to their maximal effort in 1RM, the skiers performed 10 repetitions at 60%, 8 repetitions at 70%, 6 repetitions at 80% and 3 repetitions at 90% at their subjectively estimated percentage of 1RM from their past experiences with the same exercise, respectively. The 1RM was then tested, with 1.25–2.5 kg increase at each repetition, depending on the skier's subjective assessment of the lift, until they failed to execute the exercise correctly. The rest interval between each maximal attempt was set to 2 min.

#### Submaximal steady state roller skiing test

Immediately following the 1RM test, the skiers entered the roller skiing treadmill (RL3500E, Rodby, Södertälje, Sweden) equipped with the same pair of roller skies (IDT Classic RM2, wheel type 2, IDT Solutions AS, Norway), binding system (Rottefella AS, Klokkarstua, Norway) and their own poles with special carbide tips. The participant was always secured with a safety harness hanging from the ceiling, connected to the safety brake system of the treadmill. The skiers were instrumented with a nose-clip, a two-way mouthpiece (Hans Rudolph 2700 Instr., Germany), and a heart rate (HR) monitor (RS400 HR monitor, Polar Electro Oy, Finland). Oxygen uptake ($\dot{\text{V}}$O_2_) was measured using an ergo spirometry mixing chamber system (Oxycon-Pro, Jaeger Instr., Germany), which is shown to be valid when compared against the Douglas bag method for both shorter and longer test periods in highly trained athletes (Foss and Hallén, 2005). Respiratory variables were recorded every 10 s and HR at 5 s intervals. Prior to each test, sensors were calibrated for O_2_ and carbon dioxide (CO_2_) using known gas concentrations of 16.00 and 4.90%, respectively, as well as ambient air. The inspiratory flow volume was manually calibrated using a 3-liters high-precision volume syringe (Calibration Syringe, series 5530, Hans Rudolph Instr; MO, Germany).

Following a warm-up session of 10 min (5 min of DS and 5 min of DP in randomized order) at 10.5% inclination and treadmill speed of 7 km·h^−1^, the skiers started the submaximal steady state test at the same inclination. First, the skiers performed 5 min each of DP and DS at 8 km·h^−1^ (same order as during the warm-up), with 2 min rest between the intervals. During the rest interval, the skiers reported their rating of perceived exertion (RPE) using the 6–20 point Borg scale (Borg, 1982) and blood lactate concentration (BLa) was measured using 0.2 μL capillary non-hemolyzed blood, which was sampled on a sterile single-use lancet connected to a mobile lactate analyzer (Lactate Scout +, EKF Diagnostics, Germany). Thereafter, the speed was increased to 10 km·h^−1^, and the skiers performed 5 min each of DP and DS in the same order as their 8 km·h^−1^ steady state with a 2 min rest interval, followed by the measurements of RPE and BLa.

#### Incremental test to exhaustion

Following the submaximal tests, the skiers rested for 10 min before starting the first of two ramp-tests to exhaustion. The DP and DS trials were completed in the same order as the submaximal tests. The treadmill was set to 15% inclination and a starting speed of 7 km·h^−1^, with speed being increased with 1 km·h^−1^ every minute until exhaustion. Blood lactate was measured immediately after completion of the test, and RPE was reported by the skiers 1 min after test termination. Exhaustion was defined when the skiers were not able to keep their feet in front of a marker at the side of the treadmill. Peak oxygen uptake (during DP) was defined as the mean of the three highest consecutive 10 s values of $\dot{\text{V}}$O_2_. Maximal oxygen uptake (during DS) was defined similarly, but also included a respiratory exchange ratio (RER) and BLa over 1.05 and 8 mmol·L^−1^, respectively (Howley et al., 1995). Peak (during DP) and maximal (during DS) HR was defined as the highest HR during the last minute of the test. Velocity at $\dot{\text{V}}$O_2max_ (v $\dot{\text{V}}$O_2max_) was calculated by linear interpolation using the following formula:

$$\begin{array}{l} {\text{V}}_{\text{f}}\;+\;((\text{t/60}\cdot \Delta \text{V}) \end{array}$$

With V_f_ being the speed during the last workload completed, *t* the duration of this last workload (s) and ΔV the difference in speed during the last two workloads. The skiers then rested for 20 min before starting their test to exhaustion in the other sub-technique using the same protocol. The rest interval of 20 min is the time between two final heats in a XCS sprint and was considered as sufficient time to eliminate perceived fatigue (Vesterinen et al., 2009; Andersson et al., 2017). In addition, the randomization of the starting sub-technique controlled for fatigue as a confounding factor.

### Gross efficiency

The GE of DP and DS during the submaximal tests was calculated as power divided by the metabolic rate, as provided by Sandbakk et al. (2012). The power was calculated as the sum of power against gravity (P_g_) and rolling resistance (P_f_):

$$\begin{array}{l} {\text{P}}_{\text{g}}\;+\;{\text{P}}_{\text{f}}\;=\;\text{m}\cdot \text{g}\cdot \text{v}\cdot \text{sin}(\alpha )\;+\;\text{cos}(\alpha )\cdot (\mu ) \end{array}$$

With *m*, being the system mass (body mass + equipment), *g* the gravitational constant (9.81 m·s^−2^), *v* the treadmill speed, α the treadmill inclination and μ the frictional coefficient. The frictional coefficient was set to 0.016 based on previous studies on classical roller skies (Stöggl and Holmberg, 2011, 2016a). Metabolic rate was calculated by using the $\dot{\text{V}}$O_2_ and the associated RER for VCO_2_ together with the standard conversion tables (Péronnet and Massicotte, 1991). This calculation has been used in similar studies of XC skiers (Sandbakk et al., 2012, 2016b).

### Statistical analysis

Statistical package for Social Sciences (SPSS, Version 24, International Business Machines Corporation, United States) was used to perform all statistical analysis. All results are presented as median (inter-quartile range), unless otherwise stated. To test for differences in physiological and perceptual responses between the two performance groups (VSC skiers vs. national level XC skiers), the Mann-Whitney *U*-test was applied. To test for differences between DS and DP at the same speed for all subjects pooled as well as for within-group differences, Wilcoxon's signed-rank-test was used. Alpha level of significance was set at 0.05.

## Results

### 1RM pulldown

There were no differences in 1RM pull-down performance between the VSC skiers and the national level XC skiers with respect to absolute [VSC: 90.0 (6.0) kg, national level XC: 80.0 (20.0) kg; *p* = 0.32] or relative to body weight [VSC: 1.19 (0.18), national level XC: 1.16 (0.26); *p* = 0.46] values.

### Submaximal roller skiing tests

**Figure 1:**
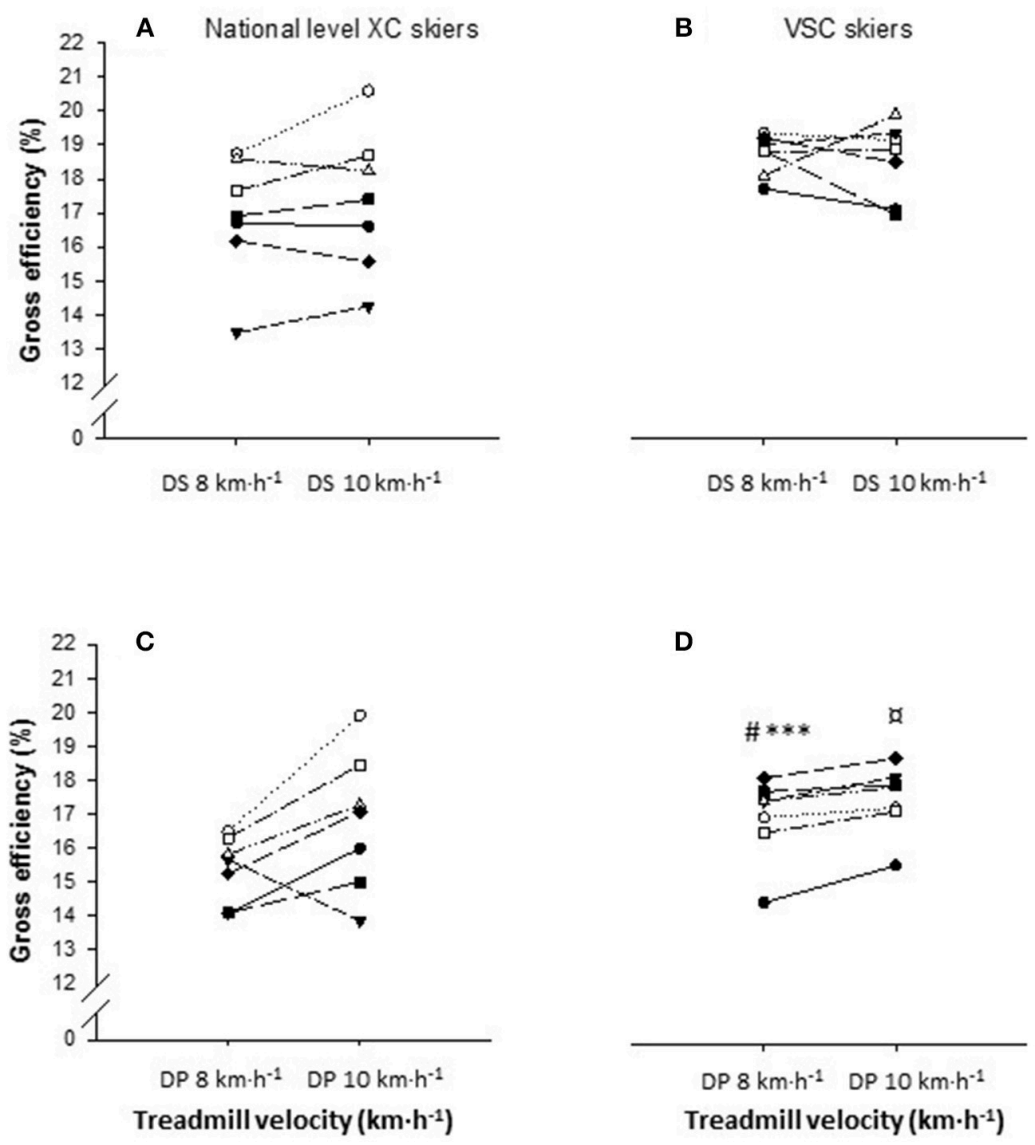
Individual plots of gross efficiency (GE) during diagonal stride (DS) at 8 km·h^−1^ and 10 km·h^−1^ for **(A)** national level cross-country (*n* = 7) and **(B)** elite Visma Ski Classics (*n* = 7) skiers, and during double poling (DS) at 8 km·h^−1^ and 10 km·h^−1^ for **(C)** national level cross-country (*n* = 7) and **(D)** elite Visma Ski Classics (*n* = 7) skiers. The inclination was set at 10.5% for both speeds and both sub-techniques. ^*^Significant higher GE for VSC skiers in DP at 8 km·h^−1^ compared with national level XC skiers, ^***^P < 0.001; ^#^Significant lower GE for VSC skiers in DP at 8 km·h^−1^ compared with DS at 8 km·h^−1^, ^#^*P* < 0.05; ^¤^Significant higher GE for VSC skiers in DP at 10 km·h^−1^ compared with DP at 8 km·h^−1^, ^¤^*P* < 0.05.

**Figure 2:**
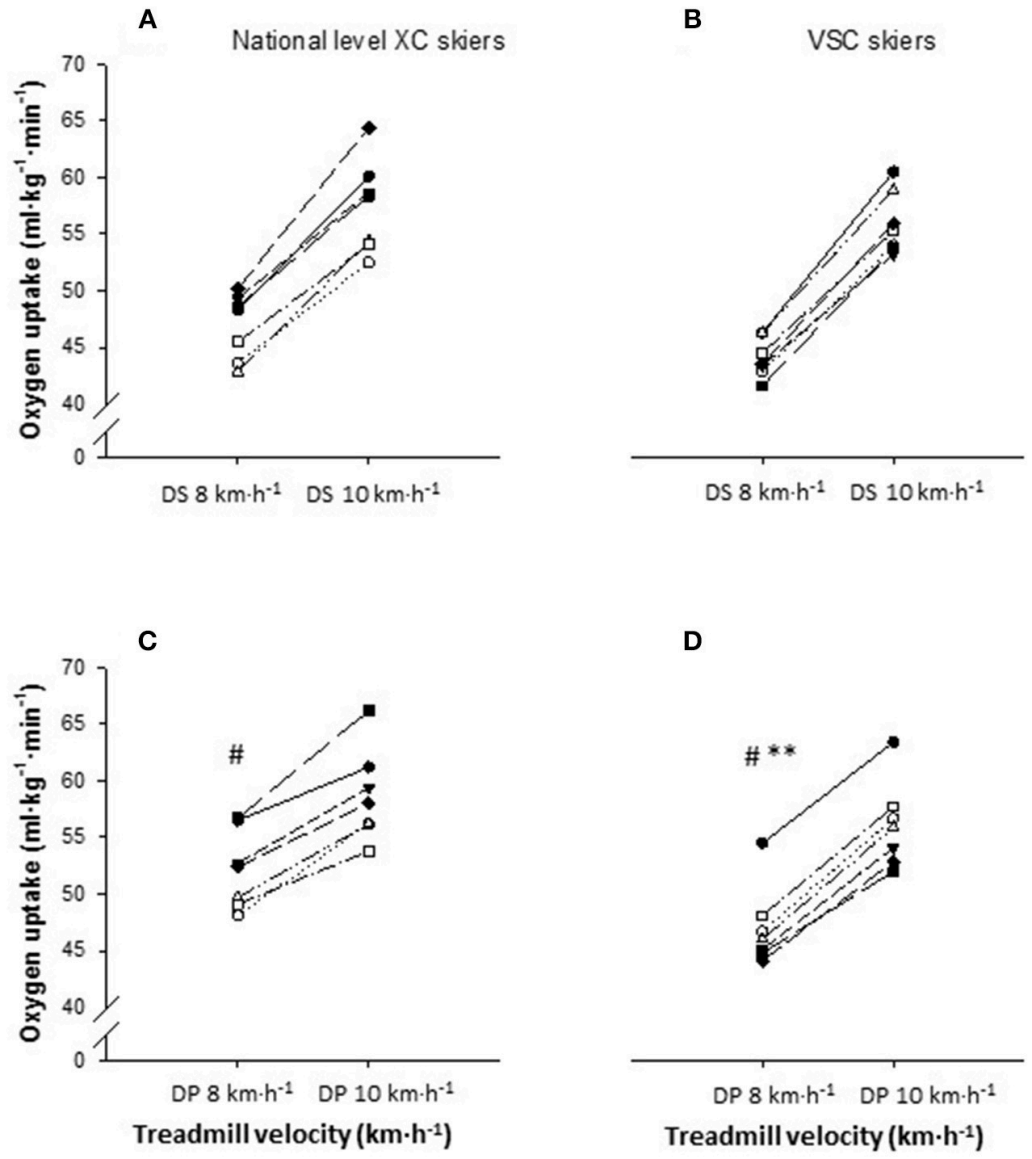
Individual plots of oxygen uptake during diagonal stride (DS) at 8 km·h^−1^ and 10 km·h^−1^ for **(A)** national level cross-country (*n* = 7) and **(B)** elite Visma Ski Classics (*n* = 7) skiers, and during double poling (DP) at 8 km·h^−1^ and 10 km·h^−1^ for **(C)** national level cross-country (*n* = 7) and **(D)** elite Visma Ski Classics (*n* = 7) skiers. The inclination was set at 10.5% for both speeds and both sub-techniques. ^*^Significant lower oxygen uptake for VSC skiers in DP at 8 km·h^−1^ compared with national level XC skiers, ^**^P < 0.01; ^#^Significant higher oxygen uptake within the same group in DP at 8 km·h^−1^ compared with DS at 8 km·h^−1^, ^#^*P* < 0.05.

All values for the submaximal roller skiing tests with respect to physiological and perceptual responses are presented in Table 2 with individual data of GE and $\dot{\text{V}}$O_2_ being illustrated in Figures 1 and 2, respectively.

**Table 2:** Physiological and perceptual responses at 8 and 10 km·h^−1^ (10.5% inclination) during submaximal diagonal stride and double poling in elite Visma Ski Classics (*n* = 7) and national level cross-country (*n* = 7) skiers.

		**DS**		**DP**	

**Parameter**		**8 km**·**h**^−1^	**10 km**·**h**^−1^	**8 km**·**h**^−1^	**10 km**·**h**^−1^
$\dot{\text{V}}$O_2_ (ml**·**kg^–1^**·**min^–1^)	All skiers pooled	45.0 (5.1)	55.6 (5.2)	48.6 (7.4)^###^	56.5 (5.8)
	VSC	43.5 (3.4)	55.3 (5.2)	46.0 (3.3)^**^^#^	55.9 (4.8)
	XC	48.3 (5.7)	58.4 (6.0)	52.4 (7.4)^#^	58.0 (5.0)
$\dot{\text{V}}$O_2_ (L**·**min^-1)^	All skiers pooled	3.4 (0.5)	4.4 (0.5)	3.7 (0.7)^###^	4.3 (0.5)
	VSC	3.4 (0.3)	4.4 (0.4)	3.6 (0.3)^#^	4.3 (0.2)
	XC	3.6 (0.7)	4.4 (0.7)	4.0 (0.6)^#^	4.3 (0.6)
% $\dot{\text{V}}$O_2max_ (DS)	All skiers pooled	61.6 (9.0)	77.1 (8.1)	67.1 (11.8)^###^	77.1 (10.8)
	VSC	56.4 (5.1)^**^	72.1 (7.4)^*^	60.5 (6.1)^**^^#^	73.1 (6.6)^*^
	XC	64.8 (5.1)	79.4 (10.9)	71.3 (7.1)^#^	80.4 (9.7)
GE (%)	All skiers pooled	17.9 (1.7)	18.0 (2.1)	16.4 (2.5)^##^	17.3 (2.4)
	VSC	18.2 (1.0)^#^	18.2 (2.0)	17.5 (1.2)^***^	17.9 (1.0)
	XC	16.9 (2.4)	17.4 (2.2)	15.6 (2.2)	17.1 (3.5)
HR (beats**·**min^–1^)	All skiers pooled	149 (28.8)	169.5 (28.5)	157.5 (29.0)^###^	169.0 (25.5)^###^
	VSC	136.0 (10.0)	156 (12.0)^*^	146.0 (13.0)^**^^#^	163.0 (13.0)^*^
	XC	162 (7.9)	182 (12.0)	173.0 (11.0)^#^	184.0 (19.0)^#^
% HR_max_ (DS)	All skiers pooled	78.9 (14.1)	91.5 (9.0)	85.1 (11.5)^###^	93.9 (7.7)^#^
	VSC	73.5 (9.3)^*^	87.4 (6.8)^*^	79.8 (5.4)^**^^#^	89.1 (2.4)^**^
	XC	85.8 (11.5)	95.0 (4.8)	91.1 (5.4)^#^	96.5 (2.4)^#^
BLa (mmol**·**L^-1^)	All skiers pooled	1.4 (0.9)	2.5 (2.6)	2.6 (2.0)^###^	4.1 (4.0)^###^
	VSC	1.2 (0.3)^*^	2.0 (1.1)^*^	1.7 (0.4)^***^^#^	2.6 (1.5)^***^^#^
	XC	2.1 (0.8)	4.7 (2.9)	3.6 (0.7)^#^	6.6 (0.9)^#^
RER	All skiers pooled	0.86 (0.04)	0.89 (0.04)	0.91 (0.05)^##^	0.94 (0.06)^###^
	VSC	0.87 (0.06)	0.90 (0.02)	0.91 (0.05)^#^	0.91 (0.04)^#^
	XC	0.85 (0.04)	0.86 (0.07)	0.93 (0.05)^#^	0.96 (0.05)^#^
RPE (6–20)	All skiers pooled	11.0 (2.5)	14.5 (2.6)	13.0 (3.0)^##^	15.5 (2.3)^##^
	VSC	11.0 (2.0)	14.0 (2.0)	13.0 (3.0)^#^	15.0 (3.0)^#^
	XC	11.0 (5.0)	15.0 (3.0)	13.0 (5.0)^#^	17.0 (2.0)^#^

Data are shown as median (inter-quartile range).

VSC, Visma Ski Classics skiers; XC, national level cross-country skiers; DS, diagonal stride; DP, double poling; $\dot{\text{V}}$O_2_, oxygen uptake; % $\dot{\text{V}}$O_2max_ (DS), percentage of maximal oxygen uptake in diagonal stride; HR, heart rate; % HR_max_ (DS), percentage of maximal heart rate in diagonal stride; GE, gross efficiency; BLa, blood lactate concentration; RER, respiratory exchange ratio; RPE, rate of perceived exertion.

* Significant difference between groups, ^*^p < 0.05,

** p < 0.01,

*** p < 0.001;

# Significant difference within groups at the same speed in different sub-techniques, ^#^p < 0.05,

## p < 0.01,

### *p < 0.001*.

For all skiers pooled, GE in DS at 8 km·h^−1^ was higher compared with DP at 8 km·h^−1^. Additionally, for GE, we also compared the difference within each sub-technique at different speeds. For all skiers pooled, the GE was higher in DP at 10 km·h^−1^ (17.3 (2.4) %) compared with DP at 8 km·h^−1^ (16.4 (2.5) %, *p* = 0.008). There were no difference between speeds in DS (*p* = 0.73). Within each of the groups separately, VSC skiers had higher GE in DP at 10 km·h^−1^ compared with DP at 8 km·h^−1^ (*p* < 0.001), whereas no differences was found for the national level XC skiers (*p* = 0.08). The VSC skiers displayed higher GE in DP at 8 km·h^−1^ compared with the national level XC skiers but not at 10 km·h^−1^.

For all skiers pooled, absolute and relative $\dot{\text{V}}$O_2_ was higher in DP at 8 km·h^−1^ compared with DS at 8 km·h^−1^, with no differences between DP and DS for relative and absolute $\dot{\text{V}}$O_2_ at 10 km·h^−1^. The VSC skiers had lower percentage of $\dot{\text{V}}$O_2max_ at all sub-techniques and speeds. The VSC skiers accumulated lower BLa, demonstrated lower absolute and relative HR at both sub-techniques and speeds compared with the national level XC skiers, except for absolute HR at 8 km·h^−1^ in DS.

As shown in Figure 1, and mentioned above, all skiers had higher GE in DP at 10 km·h^−1^ (range VSC skiers: 16–19%, range national level XC skiers: 14–20%) compared with 8 km·h^−1^ (range VSC skiers: 15–18%, range national level XC skiers: 14–17%). The range in GE for VSC skiers in DS at 10 km·h^−1^ was 17–19% and at 8 km·h^−1^ the range was 17–19%. For the national level XC skiers, the range in GE in DS at 10 km·h^−1^ was 14–21% and at 8 km·h^−1^ in DS the range was 14–19%.

As shown in Figure 2, the range in $\dot{\text{V}}$O_2_ for VSC skiers in DS at 10 km·h^−1^ was 53–61 ml·kg^−1^·min^−1^ and at 8 km·h^−1^ the range was 42–46 ml·kg^−1^·min^−1^. The range for the national level XC skiers in DS at 10 km·h^−1^ was 53–64 ml·kg^−1^·min^−1^, whereas the range at 8 km·h^−1^ was 43–50 ml·kg^−1^·min^−1^. The range for the VSC skiers in DP at 10 km·h^−1^ was 53–63 ml·kg^−1^·min^−1^ and at 8 km·h^−1^ the range was 44–55 ml·kg^−1^·min^−1^. The range for the national level XC skiers in DP at 10 km·h^−1^ was 54–66 ml·kg^−1^·min^−1^, whereas the range at 8 km·h^−1^ was 48–57 ml·kg^−1^·min^−1^.

### Test to exhaustion

The physiological and perceptual responses for the maximal tests until exhaustion are shown in Table 3. The $\dot{\text{V}}$O_2peak_ in DP was 6.8 ml·kg^−1^·min^−1^ lower compared with $\dot{\text{V}}$O_2max_ in DS in both VSC and national level XC skiers. There was no difference in $\dot{\text{V}}$O_2peak_ DP/$\dot{\text{V}}$O_2max_ DS-ratio between VSC skiers (91.1 (0.1) %) and national level XC skiers (89.9 (0.1) %).

**Table 3:** Physiological and perceptual responses at 15% inclination in the test to exhaustion in diagonal stride and double poling in elite Visma Ski Classics (*n* = 7) and national level cross-country (*n* = 7) skiers.

**Parameter**		**DS**	**DP**
$\dot{\text{V}}$O_2_ (ml**·**kg^–1^**·**min^–1^)	All skiers pooled	74.8 (3.8)^###^	67.9 (6.1)
	VSC	76.0 (6.6)^#^	71.9 (7.5)
	XC	74.5 (5.1)^#^	66.5 (9.1)
$\dot{\text{V}}$O_2_ (L**·**min^–1^)	All skiers pooled	5.8 (1.1)^###^	5.2 (0.9)
	VSC	5.9 (0.7)^#^	5.4 (0.5)
	XC	5.3 (1.3)^#^	4.5 (0.9)
HR (beats**·**min^–1^)	All skiers pooled	185.0 (11.5)^##^	181.5 (17.3)
	VSC	185.0 (8.0)^#^	176.0 (10.0)
	XC	190.0 (22.0)	190.0 (13.0)
TTE (s)	All skiers pooled	372.5 (93.3)^###^	238.5 (65.3)
	VSC	419.0 (76.0)^#^^*^	261.0 (51.0)^***^
	XC	343.0 (58.0)^#^	202.0 (35.0)
v$\dot{\text{V}}$O_2max_ (km**·**h^–1^)	All skiers pooled	12.2 (1.6)^###^	10.0 (1.09)
	VSC	13.0 (1.3)^#^^*^	10.4 (0.8)^***^
	XC	11.7 (1.0)^#^	9.4 (0.6)
BLa (mmol**·**L^–1^)	All skiers pooled	10.4 (1.9)	10.4 (1.8)
	VSC	10.4 (3.1)	10.2 (1.6)
	XC	10.4 (1.4)	11.1 (1.6)
RER	All skiers pooled	1.10 (0.07)	1.0 (0.07)
	VSC	1.10 (0.06)^*^	1.05 (0.09)
	XC	1.03 (0.05)	1.03 (0.07)
RPE (6–20)	All skiers pooled	20.0 (0.5)	20.0 (1.3)
	VSC	20.0 (2.0)	20.0 (2.0)
	XC	20.0 (0.0)	20.0 (1.0)

Data are shown as median (inter-quartile range).

VSC, Visma Ski Classics skiers; XC, national level cross-country skiers; DS, diagonal stride; DP, double poling; $\dot{\text{V}}$O_2_, oxygen uptake; HR, heart rate; % HR_max_ (DS), percentage of maximal heart rate in diagonal stride; BLa, blood lactate concentration; RER, respiratory exchange ratio; RPE, rate of perceived exertion.

* Significant difference between groups, ^*^P < 0.05, ^**^P < 0.01,

*** P < 0.001;

# Significant difference within groups in different sub-techniques, ^#^P < 0.05,

## P < 0.01,

### *P < 0.001*.

For both groups, time to exhaustion (TTE) was longer in DS compared with DP, and the VSC skiers had longer TTE in DS and DP compared with the national level XC skiers. Consequently, the velocity at exhaustion was higher in DS compared with DP for both groups, and the VSC skiers had higher velocity compared with the national level XC skiers in DP but not in DS. Finally, the RER was higher for the VSC skiers compared with the national level XC skiers in DS but not in DP.

## Discussion

The main findings of this study were: (1) VSC skiers displayed higher GE in DP at 8 km·h^−1^ compared with national level XC skiers, while this was not found at 10 km·h^−1^, (2) VSC skiers had lower BLa and HR (both absolute and relative percentage of HR_max_) at both submaximal speeds, (3) the $\dot{\text{V}}$O_2max_ in DS was higher compared with the $\dot{\text{V}}$O_2peak_ in DP, but there were no differences between groups in both sub-techniques, (4) the $\dot{\text{V}}$O_2peak_/ $\dot{\text{V}}$O_2max_-ratio did not differ between the two groups, and (5) VSC and national level XC skiers showed similar strength levels in regard of 1RM pulldown.

### One repetition maximum pulldown

VSC skiers performed similar in the 1RM cable pulldown exercise compared with the national level XC skiers in the present study, which is consistent with one previous study using the same exercise (Hegge et al., 2015). However, comparisons with Hegge et al. (2015) should be done with caution due to uncertainty regarding testing protocol differences. On the one hand, considering similar performance in 1RM pulldown between the groups, VSC skiers seem to meet no further maximal strength requirements for the upper body for speed and accelerations during competition compared with Olympic distance XC skiers. On the other hand, one may speculate whether todays VSC skiers have peaked in terms of 1RM strength for performance improvements. For example, maximal upper body strength training was reported to improve work economy in DP in female XC skiers (Hoff et al., 1999), and 1RM was shown to be important for DP performance in female XC skiers (Østerås et al., 2016). Although a similar expectation of results is likely for men, this is yet to be elucidated and evaluated (Sandbakk and Holmberg, 2017).

### Submaximal work rates

For the pooled analysis, it was demonstrated that GE was higher in DS compared with DP at 8 km·h^−1^. On the one hand, this might be explained by the specificity of training. In traditional Olympic distance skiing, ~50% of the race time is spent uphill (Andersson et al., 2010; Bolger et al., 2015; Sandbakk et al., 2016a; Sandbakk and Holmberg, 2017), which may force skiers to employ DS for larger parts of the race. On the other hand, in contrast to Olympic distance races, VSC races—except for the “Reistadløpet”—consist mainly of flat and moderate steep terrain favoring the use of the DP sub-technique, which suggest that GE would be higher in DP compared with DS for the VSC skiers. However, no such difference was found, which suggest the higher GE in DS may be physiologically explained. Although the DP sub-technique has evolved recently (Holmberg et al., 2006; Stöggl and Holmberg, 2011, 2016b; Holmberg, 2015), DS is a combined arm and leg exercise resulting in higher peripheral muscle mass contribution and thereby oxygen extraction compared with DP (Stenberg et al., 1967; Holmberg, 2015).

Further, GE in DS at 8 km·h^−1^ and 10 km·h^−1^ was similar for both groups. This suggests that skiers can increase their speed while moving uphill without any lower efficiency during DS execution, which may be an effective strategy for spreading a group of, or move away from, competitors during a race. The larger amount of DS training in the XC skiers in contrast to substantially higher DP training in the VSC skiers (see Table 1) might be the reason for the similar GE results in the two submaximal speeds. It should be noted that the national XC skiers in this study performed ~600 h of training annually (see Table 1), which is lower compared with the VSC skiers in the present study and Olympic distance winners in previous studies (~800–900 h) (Sandbakk and Holmberg, 2014, 2017).

VSC skiers displayed higher GE in DP at 8 km·h^−1^ compared with national level XC skiers. While DS is usually performed over moderate to steep inclines, DP is usually performed over flat- to medium-steep terrain (Holmberg et al., 2005; Stöggl and Holmberg, 2016b). The explanation for the higher GE values in VSC skiers compared with national level XC skiers can be attributed toward the higher training volumes in VSC skiers, which is similar to Olympic distance XC skiers (Sandbakk and Holmberg, 2014, 2017). Furthermore, >50% of their training is performed in DP while for the national level XC skiers of the current study only ~15% of the training volume is performed in DP (see Table 1). Finally, the duration of VSC races is several hours, and as VSC races generally favor DP employment for most parts of the tracks, VSC skiers execute the DP sub-technique in competition for long durations. Unfortunately, the national level skiers‘ BLa at 10 km·h^−1^, in particular in DP, indicates that this speed was not submaximal, which makes the national level XC skiers' GE values for DP at 10 km·h^−1^ invalid. Thus, comparisons in regard of high speed between groups are unavailable due to the calculation criteria for GE (Sandbakk et al., 2012).

The inter-individual difference in GE seem similar in VSC and national XC skiers in all submaximal speeds and sub-techniques, and varies from 14–20% (range diff.: 2–4%). This is similar to the GE range found in elite cyclists (19–24%) (Joyner and Coyle, 2008), however, it is smaller compared with a previous study of elite XC skiers employing DS at 8.7% inclination (range diff.: 10%) (Ainegren et al., 2013). Thus, the observed inter-individual difference in GE in this present study can be considered small.

The $\dot{\text{V}}$O_2_ requirements and HR were lower in DS compared with DP, which is in contrast with an earlier study, who reported similar $\dot{\text{V}}$O_2_ and HR in DS and DP (Hoffman et al., 1994). The study by Hoffman et al. (1994) included both men and women, whereas the current study investigated only men. This, together with the recent developments in the DP sub-technique and the increased employment of the DP sub-technique may explain the inconsistent results.

BLa were lower for DS compared with DP, which is consistent with previous findings (Van Hall et al., 2003; Rud et al., 2014). As DS results in higher BLa uptake in muscles due to the larger involvements of the lower body, DP seems to accumulate higher BLa values compared with DS (Van Hall et al., 2003).

### Performance in maximal tests to exhaustion

The VSC skiers displayed similar $\dot{\text{V}}$O_2max_ (DS) and $\dot{\text{V}}$O_2peak_ (DP) compared with the national level XC skiers. This is in contrast to previous findings in sprint skiers (Sandbakk et al., 2010, 2011b) and Olympic distance skiers (Bergh, 1987) comparing world-class level with national level skiers. One explanation may be that VSC skiers employ the DP sub-technique to exhaustion in competition, making employment of DS to exhaustion unfamiliar and thereby impairing their oxygen delivery- and utilization capacity while employing DS at high speeds. As DP execution is familiar for the VSC skiers and they perform higher volumes of this sub-technique compared with the national level XC skiers (see Table 1), one would expect an opposite results in $\dot{\text{V}}$O_2peak_ in DP between groups where the VSC skiers display higher $\dot{\text{V}}$O_2peak_ compared with the national level XC skiers. However, no such difference was found. This may suggest a lack of statistical power in the present study for this variable. Nonetheless, the aerobic power of the VSC skiers in the present study can be classified as high (relative to body weight: 76.0 (6.6) ml·kg^−1^·min^−1^, absolute: >5.9 (0.7) L·min^−1^, highest: 7.0 L·min^−1^) and comparable to previous findings in Olympic distance winners (Ingjer, 1991; Holmberg et al., 2007; Bergh and Forsberg, 2008; Sandbakk and Holmberg, 2014; Tønnessen et al., 2014, 2015).

Both VSC and national level XC skiers displayed higher $\dot{\text{V}}$O_2_ at maximal power during DS compared with DP, which confirms previous findings (Saltin, 1997; Holmberg et al., 2007; Sandbakk et al., 2014). As stated above, a combined arm and leg exercise is suggested to result in higher oxygen extraction by muscles as more muscle mass in the periphery is used (Stenberg et al., 1967; Holmberg, 2015). In addition, recent findings also suggest a mechanical hindrance of oxygen extraction while employing DP (Stöggl et al., 2013; Björklund et al., 2015). As an employment of DS may demand a larger proportion of muscle mass compared with DP and lower mechanical hindrance of oxygen extraction, this is likely to result in higher stroke volume, arterial desaturation and maximal ventilation in DS at maximal work rates compared with DP (Holmberg et al., 2007; Stöggl et al., 2013; Björklund et al., 2015).

Further, the $\dot{\text{V}}$O_2peak_/$\dot{\text{V}}$O_2max_-ratio presented in this study is high, which demonstrates and confirms the development of ~90% utilization reported previously (Saltin, 1997). Furthermore, some have suggested this ratio may reach >95% if the development continues (Stöggl et al., 2010; Holmberg, 2015; Sandbakk and Holmberg, 2017). In fact, two participants in the present study displayed exceptionally high ratios of 97%—with one being among the best VSC skiers in the world—which indeed illustrates the ability of human development of upper body capacity, as suggested earlier (Stöggl et al., 2010; Sandbakk and Holmberg, 2017). How this is obtained remains unclear (Holmberg, 2015). VSC skiers report high volumes of upper body training (see Table 1), and an earlier investigation reported that high-volume, low-intensity training improved arm crank $\dot{\text{V}}$O_2peak_ (Boushel et al., 2014).

Considering the high volume of DP execution by the VSC skiers in both competition and training, as well as the observed DP sub-technique developments within the past years, one may speculate if similar GE performance and $\dot{\text{V}}$O_2max_ in DS and $\dot{\text{V}}$O_2peak_ in DP for VSC skiers could be possible in the future.

By comparing the difference in overall performance in tests between the two groups, VSC skiers had higher GE in DP at 8 km·h^−1^, and lower BLa and HR at both submaximal speeds in both sub-techniques, a longer time to exhaustion (TTE) but not higher aerobic power. Longer TTE and higher GE is in agreement with previous findings in XC sprint skiers comparing world-class level with national level, however, in contrast to our study, sprint XC skiers also differ in aerobic power (Sandbakk et al., 2010, 2011b). It was previously suggested if aerobic power is similar between two groups of different performance levels, the two most plausible factors differing these athletes are oxygen utilization and efficiency (Joyner and Coyle, 2008).

### Limitations

This study may have suffered from low statistical power in some variables. For example, the VSC skiers performed non-significantly (*p* = 0.32) ~ 12.5% better in median 1RM cable pulldown. Moreover, $\dot{\text{V}}$O_2peak_ in DP was not different between VSC and national level XC skiers. A larger sample size may have revealed more potential significant differences. At the same time, elite athletes are few and are sometimes hard to recruit for intensive tests and/or experiments. As these athletes (especially the VSC skiers) are at the peak level of performance in their sport, low sample sizes may sometimes be an acceptable limitation to understand mechanisms of elite performance. Nevertheless, the low sample size should be considered as the major limitation in this study. Thus, this study could be considered a pilot study to inform future research.

The protocol for submaximal tests at 10.5% inclination and 10 km·h^−1^ may have been too demanding for the national level XC skiers. Based on BLa values for national level XC skiers at 10 km·h^−1^, including a lower speed, for instance 6 km·h^−1^, would have been beneficial. Nevertheless, the difference in BLa and HR may reflect a difference in performance level.

As execution of DP is to a great extent performed in flat terrain, a submaximal stage of 10.5 % incline may not be suitable for DP employment. Thus, a submaximal stage of flat terrain may have revealed further interesting results regarding GE values, in particular for the VSC skiers. However, our primary aim was to compare the two sub-techniques at the same external conditions (e.g., speed and incline), and the inclusion of two submaximal stages of lower incline could have excluded some participants due to time consumption. Moreover, as the employment of DP have increased in recent years, DP is also performed in moderate to steep terrain, making DP employment at higher inclinations applicable.

Unfortunately, we did not possess any equipment to measure friction of the roller skies used in this study. Therefore, the frictional coefficient used in the GE calculation was adapted from other studies using classical rollers skiers (Stöggl and Holmberg, 2011, 2016a). To our knowledge, no study has employed the IDT solutions classical roller skies in experimental settings and reported the rolling resistance of the skies. Thus, some caution should be taken when interpretation the GE results in this present study. However, this possible systematic error should be rather low due to the relatively steep incline of 10.5% making the relative influence of rolling resistance on the total power output relatively low.

Other studies focusing on performance level differences in XCS, compared groups of similar competition preferences (Bergh, 1987; Ingjer, 1991; Sandbakk et al., 2010, 2011b; Stöggl et al., 2010; Welde et al., 2017). In our study, the VSC skiers were compared with national level XC skiers competing in the Olympic distances arranged by the FIS. Finding more suitable matches for the VSC skiers in regard to competition preference would make the comparisons between groups more valuable. On the other hand, considering the sub-techniques and sport to be equal, the main differences between the groups are the competition duration and the distribution of training modalities. Thus, comparing skiers competing primarily in Olympic distances with VSC distances is both justified and applicable.

This study investigated males exclusively. There are fewer studies investigating female's characteristics in XCS, and to our knowledge, no study have investigated this in females who compete in the VSC. Future perspectives may reflect potential differences between sexes.

## Conclusion

VSC skiers show many similar characteristics as Olympic distance skiers. The main differences were lower cardiorespiratory and metabolic responses at submaximal speeds as well as increased time to exhaustion. These findings suggest the main differences to be efficiency and oxygen extraction by small muscle mass between VSC skiers and national level XC skiers. The peak oxygen uptake/maximal oxygen uptake-ratio seems to move toward >95% for specialized skiers who apply high volumes of DP. Future work should emphasize comparisons of world-class XC skiers of different racing preferences and both sexes to reflect potential differences between these skiers.

## Author's note

A part of this research article was presented at the European College of Sport Science annual congress in Dublin 2018 as a poster presentation. ES was the presenting author with the title: Gross efficiency and peak oxygen uptake in Visma ski classics athletes.

## Author contributions

ES: in charge of the writing process. TE, GM, and TOT: test protocol design and data collection. SP, SAP, and KH: manuscript writing. BW: statistics, manuscript writing and test protocol design. TS: manuscript writing and test protocol design.

### Conflict of interest statement

The authors declare that the research was conducted in the absence of any commercial or financial relationships that could be construed as a potential conflict of interest.
